# Head and neck oncology management in the time of COVID-19: results of a head and neck cancer center

**DOI:** 10.1007/s00432-023-05122-1

**Published:** 2023-07-08

**Authors:** Silvia Heckel, Christopher Bohr, Johannes Meier, Julia Maurer, Julian Kuenzel, Karolina Mueller, Oliver Koelbl, Torsten Reichert, Veronika Vielsmeier, Isabella Gruber

**Affiliations:** 1 University of Regensburg, Universitätsstraße 31, 93053 Regensburg, Germany; 2 Department of Otorhinolaryngology, Head and Neck Surgery, University Hospital Regensburg, Franz-Josef-Strauss Allee 11, 93053 Regensburg, Germany; 3 Department of Maxillofacial Surgery, University Hospital Regensburg, Franz-Josef-Strauss Allee 11, 93053 Regensburg, Germany; 4 University Cancer Center Regensburg, Franz-Josef-Strauss Allee 11, Regensburg, Germany; 5 Center for Clinical Studies, University Hospital Regensburg, Franz-Josef-Strauss Allee 11, 93053 Regensburg, Germany; 6 Department of Radiation Oncology, University Hospital Regensburg, Franz-Josef-Strauss Allee 11, 93053 Regensburg, Germany

**Keywords:** Pandemic, COVID-19, Cancer care, Oncologic surgery, Head and neck cancer, Oncological performance

## Abstract

**Purpose:**

Given the concerns about the effects of the COVID-19 pandemic on cancer care, we analyzed the treatment quality of the head and neck cancer center Regensburg before and throughout 2 years of the pandemic. We included data of 3 years to reflect the extended pandemic period as new developments continued to influence its course.

**Methods:**

This retrospective review included all patients diagnosed with head and neck cancer in 2019, 2020, and 2021 who had not started treatment elsewhere prior to being referred to the head and neck cancer center. We compared tumor characteristics and times to therapy of patients diagnosed before COVID-19 in 2019 (n = 253), during COVID-19 in 2020 (n = 206), and in a phase of partial normalization in a persistent pandemic situation in 2021 (n = 247).

**Results:**

Our data revealed no decrease in diagnoses or drift in stages toward more advanced stages. There was an increased percentage of diagnoses confirmed at the head and neck cancer center from 2019 (57.3%) to 2020 (68.0%) and to 2021 (65.6%) compared to confirmation at other institutions (2019, 42.7%; 2020, 32.0%; 2021, 34.4%; *P* = 0.041). Surgery and radiotherapy were performed with the same frequency. The median days between diagnosis and surgery were decreased in 2020 (19.5 days; *P* = 0.049) and 2021 (20.0 days; *P* = 0.026) in comparison to 2019 (23 days). The days to radiotherapy were not affected.

**Conclusion:**

The data indicate a consistent oncological performance for head and neck cancer patients in all waves of the pandemic and thereafter without a decrease in diagnoses or shift in stages.

## Introduction

On the 11th of March 2020, the World Health Organization (WHO) declared the coronavirus disease 2019 (COVID-19) outbreak a pandemic (WHO 2020). Following this declaration and the spread of COVID-19 cases, the German government started implementing restrictions to keep the healthcare system from getting overwhelmed and to control the situation as best as possible. As of 16th of March 2020 non-essential stores were ordered closed by the Bavarian government (Allgemeinverfügung des Bayerischen Staatsministerium 2020), followed by a country-wide stay-at-home order going into effect on 22nd of April 2020. Additionally, elective surgery and treatment requiring an inpatient stay were to be postponed keeping resources available for COVID-19 patients. Patients in need of timely treatment including oncologic patients were exempt from these regulations, as the swift initiation of treatment in these cases remained a priority.

Preliminary data from the University of Texas M. D. Anderson Cancer Center (Kiong et al. 2021) comparing head and neck cancers over a 6-week period before and during the COVID-19 pandemic revealed a 25% reduction in newly diagnosed head and neck malignancies in the first wave of the COVID-19 pandemic. Since the COVID-19 pandemic affected different nations and geographical areas to different degrees, the results of this landmark study are not necessarily indicative of the situation in Germany. Some literature suggests that the impact of COVID-19 was limited to certain tumor entities and not all diagnoses were equally affected by the pandemic in Germany (Voigtländer et al. 2023; Jacob et al. 2022; Piontek et al. 2021). Jacob et al. (2022) compared the number of patients diagnosed with cancer in general and specialized practices in Germany in April 2020-March 2021 (COVID-19 era) and April 2019-March 2020 (pre-COVID-19 era). Jacob et al. (2022) found a significant decrease in the number of patients diagnosed with skin cancer (12.8% reduction), not of patients diagnosed with lip, oral cavity, and pharynx cancer. Voigtländer et al. (2023) analyzed the impact of the pandemic on reported cancer cases in Bavaria and compared cancer cases of the pre-pandemic period (March 2019 to February 2020) and pandemic period (March 2020 to February 2021).Significant reductions were found for colon, rectum, and skin/melanoma (> 10% reduction) without a decrease in lip, oral cavity, and pharynx cancers. The majority of these studies were restricted to data obtained in the first wave of the pandemic and up to February 2021 limiting the generalizability of the results to the whole period of the pandemic. The results of the present study are innovative compared to previous studies as we include data of 3 years (2019, 2020, and 2021) to examine how the treatment dynamics may have changed not just in comparison to pre-pandemic conditions (2019) but also in 2021. The year 2021 was a time of partial normalization as the European Medicine Agency (EMA) authorized the first vaccines in December 2020 which constituted a new variable in the course of the pandemic. Including complete data from 2021 offers insight into the effects of the pandemic beyond the first waves and gives us an idea regarding the adaptability of the healthcare system to drastically changed circumstances. Additionally, we contribute to the existing literature by showing data from a University Cancer Center known to be of high quality according to international standards.

## Materials and methods

### Data collection

This retrospective review included all patients diagnosed with head and neck cancer in 2019, 2020, and 2021 who had not started treatment elsewhere prior to being referred to our head and neck cancer center and were above 18 years of age. Thus this study only includes patients diagnosed who received primary treatment at our head and neck cancer center. We compared data from three consecutive years to verify if the COVID-19 pandemic influenced the number of diagnoses and the oncological treatment beyond the first waves. Control group A included patients diagnosed from January 2019 to December 2019 (pre-pandemic study group A, year 2019). Study group B included patients diagnosed from January 2020 to December 2020 during the beginning of the COVID-19 pandemic (pandemic study group B, year 2020). Patients of study group C were diagnosed between January 2021 and December 2021 during a phase of partial normalization in a persistent pandemic situation (group C, year 2021). Data were extracted from the electronic charts of the University Cancer Center Regensburg (UCC-R). The UCC-R records and updates patient and tumor characteristics for cancers diagnosed in the eastern part of Bavaria. Data were confirmed by manual chart review of medical charts of the University Hospital Regensburg. The choice of treatment (surgery and/or radiotherapy) was based on the interdisciplinary tumor board/physicians´ discretion and dependent on the tumor stage and/or the presence of comorbidities. All of the patients were staged according to the 8th Edition of AJCC (American Joint Committee on Cancer) TNM Classification. We used the 10th revision of the International Classification of Diseases (ICD). The analysis included cancers of the base of the tongue (ICD-C01), cancers of other and unspecified parts of the tongue (ICD-C02), cancers of the gingiva (ICD-C03), cancers of the floor of the mouth (ICD-C04), cancers of the palate (ICD-C05), cancers of other and unspecified parts of the mouth (ICD-C06), cancers of the parotid glands (ICD-C07), cancers of other and unspecified large salivary glands (submandibular glands, sublingual glands; ICD-C08), cancers of the tonsils (ICD-C09), oropharyngeal cancers (ICD-C10), nasopharyngeal cancers (ICD-C11), cancers of the piriform sinus (ICD-C12), hypopharyngeal cancers (ICD-C13), cancers of other and imprecisely defined localizations of the lips, mouth, and pharynx (ICD-C14), cancers of the nasal cavity and middle ear (ICD-C30), cancers of the paranasal sinus (ICD-C31) and laryngeal cancers (ICD-C32).

The primary endpoint was the time to treatment initiation (surgery and/or radiotherapy). Secondary endpoints were tumor characteristics, the number of diagnoses (primary cancer, non-primary cancer/relapse) and diagnosing departments (head and neck cancer center Regensburg, other departments). Variables included patient age, sex, date of diagnosis (date of histological confirmation of cancer), diagnosing department (head and neck cancer center Regensburg, other departments), type of cancer (primary cancer, non-primary cancer/relapse), ICD-10 codes, UICC/TNM classification, date of surgery, date of the beginning of radiotherapy (definitive/primary radiotherapy, adjuvant radiotherapy), and intention of radiotherapy (curative, palliative). We calculated diagnosis-treatment intervals as days between the date of diagnosis through histological confirmation of cancer and initiation of therapy (either surgery or definitive radio/chemotherapy), and days between surgery and adjuvant radio/chemotherapy. The local Ethics Board of the University of Regensburg approved this study (Number 22-2900-104, date 04/13/2022).

### Statistical analysis

Characteristics were presented as median and interquartile range (IQR) for continuous variables and as absolute and relative frequencies for categorical variables. The Kruskal–Wallis-Test was used for comparisons of continuous variables, and the chi-square test of independence for categorical variables. All *P* values were two-sided and *P* values < 0.05 were considered significant. Statistical analysis was performed using SPSS 28.0 (SPSS Inc., Chicago, IL, USA).

## Results

A total of 706 patients with head and neck cancers were diagnosed between 2019 and 2021 (year 2019, *n* = 253; year 2020, *n* = 206; year 2021, *n* = 247). Forty-six patients were diagnosed with cancers of the base of the tongue (ICD-C01), 104 patients with cancers of other and unspecified parts of the tongue (ICD-C02), 89 patients with cancers of the gingiva (ICD-C03), 76 patients with cancers of the floor of the mouth (ICD-C04), 44 patients with cancers of the palate (ICD-C05), and 22 patients with cancers of other and unspecified parts of the mouth (ICD-C06). There were 25 cancers of the parotid gland (ICD-C07), 8 cancers of other and unspecified large salivary glands (submandibular glands, sublingual glands; ICD-C08), 62 cancers of the tonsils (ICD-C09), 18 oropharyngeal cancers (ICD-C10), 10 nasopharyngeal cancers (ICD-C11), 22 cancers of the piriform sinus (ICD-C12), 30 hypopharyngeal cancers (ICD-C13), 7 cancers of other and imprecisely defined localizations of the lips, mouth, and pharynx (ICD-C14), 43 cancers of the nasal cavity and middle ear (ICD-C30), 21 cancers of the paranasal sinus (ICD-C31) and 79 laryngeal cancers (ICD-C32).

The patient characteristics are presented in Table 1. Baseline characteristics were similar between the groups. The three groups did not differ with respect to sex, median age, or tumor type (primary cancer vs. relapse). Comparing the 3 years, the distributions of diagnoses over the quarters were similar. There was an increased percentage of diagnoses confirmed at the head and neck cancer center Regensburg from 2019 (57.3%) to 2020 (68.0%) and to 2021 (65.6%) compared to confirmation at other institutions (2019, 42.7%; 2020, 32.0%; 2021, 34.4%; *P* = 0.041). The distribution of the UICC classifications did not differ between the 3 years. The patients did not present with higher T stages, more loco-regional affection of lymph nodes (N stages), or metastatic diseases (M stages) during the pre-pandemic year 2019, the pandemic year 2020, or the following year 2021 (Table 1).

**Table 1:** Patient characteristics

Characteristics	Group A, year 2019, pre-COVID-19^a^ (*n* = 253)	Group B, year 2020, COVID-19^b^ (*n* = 206)	Group C, year 2021, COVID-19^c^ (*n* = 247)	*p* value
Patient age, years				0.803
Median (interquartile range)	63.0 (56.0–71.0)	63.0 (56.0–72.0)	63.0 (56.0–71.0)	
Gender				0.572
Female	66 (26.1%)	62 (30.1%)	65 (26.3%)	
Male	187 (73.9%)	144 (69.9%)	182 (73.7%)	
Type of cancer				0.618
Primary cancer	237 (93.7%)	192 (93.2%)	226 (91.5%)	
Non-primary cancer/relapse	16 (6.3%)	14 (6.8%)	21 (8.5%)	
Number of diagnoses				0.233
1st quarter	67 (26.5%)	57 (27.7%)	61 (24.7%)	
2nd quarter	55 (21.7%)	38 (18.4%)	61 (24.7%)	
3rd quarter	75 (29.6%)	47 (22.8%)	63 (25.5%)	
4th quarter	56 (22.1%)	64 (31.1%)	62 (25.1%)	
Diagnosing departments				0.041
Head and neck cancer center	145 (57.3%)	140 (68.0%)	162 (65.6%)	
Other	108 (42.7%)	66 (32.0%)	85 (34.4%)	
UICC classification				0.567
I	77 (30.4%)	63 (30.6%)	62 (25.1%)	
II	36 (14.2%)	34 (16.5%)	51 (20.6%)	
III	35 (13.8%)	32 (15.5%)	38 (15.4%)	
IVA	80 (31.6%)	54 (26.2%)	65 (26.3%)	
IVB	21 (8.3%)	12 (5.8%)	21 (8.5%)	
IVC	2 (0.8%)	6 (2.9%)	8 (3.2%)	
Missing values	2 (0.8%)	5 (2.4%)	2 (0.8%)	
Pathologic/clinical T classification				0.615
Pathologic	199 (78.7%)	152 (73.8%)	191 (77.3%)	
Clinical	50 (19.8%)	47 (22.8%)	49 (19.8%)	
Missing values	4 (1.6%)	7 (3.4%)	7 (2.8%)	
T stage				0.787
Early stage (T1, T2)	147 (58.1%)	126 (61.2%)	148 (59.9%)	
Advanced stage (T3, T4)	102 (40.3%)	78 (37.9%)	92 (37.2%)	
Missing values	4 (1.6%)	2 (1.0%)	7 (2.8%)	
Pathologic/clinical N classification				0.981
Pathologic	143 (56.5%)	115 (55.8%)	137 (55.5%)	
Clinical	108 (42.7%)	90 (43.7%)	106 (42.9%)	
Missing values	2 (0.8%)	1 (0.5%)	4 (1.6%)	
N stage				0.337
Node negative (N0)	155 (61.3%)	131 (63.6%)	141 (57.1%)	
Node positive (N1,2,3)	96 (37.9%)	74 (35.9%)	105 (42.5%)	
Missing values	2 (0.8%)	1 (0.5%)	1 (0.4%)	
M stage				0.129
No metastases (M0)	249 (98.4%)	199 (96.6%)	236 (95.5%)	
Metastases (M1)	2 (0.8%)	6 (2.9%)	8 (3.2%)	
Missing values	2 (0.8%)	1 (0.5%)	3 (1.2%)	

^a^Year 2019, pre-pandemic control group

^b^Year 2020, pandemic study group

^c^Year 2021, a phase of partial normalization in a persistent pandemic situation

Table 2 presents the frequencies of surgery and radiotherapy during the years 2019, 2020, and 2021.

**Table 2:** Frequencies of surgery and radiotherapy during the years 2019, 2020 and 2021

Characteristics	Group A, year 2019, pre-COVID-19^a^ (*n* = 253)	Group B, year 2020, COVID-19^b^ (*n* = 206)	Group C, year 2021, COVID-19^c^ (*n* = 247)	*P* value
Surgery				0.675
Yes	204 (80.6%)	161 (78.2%)	201 (81.4%)	
No	49 (19.4%)	45 (21.8%)	46 (18.6%)	
Radiotherapy^d^				0.595
Yes	76 (30.0%)	71 (34.5%)	78 (31.6%)	
No	177 (70.0%)	135 (65.5%)	169 (68.4%)	

^a^Year 2019, pre-pandemic control group

^b^Year 2020, pandemic study group

^c^Year 2021, a phase of partial normalization in a persistent pandemic situation

^d^Primary/definitive and adjuvant radiotherapy

The distributions of primary/definitive vs. adjuvant radiotherapy and the intention of radiotherapy (curative vs. palliative) were similar between the three years (Table 3).

**Table 3:** Radiotherapy during the years 2019, 2020 and 2021

Characteristics	Group A, year 2019, pre-COVID-19^a^ (*n* = 76)	Group B, year 2020, COVID-19^b^ (*n* = 71)	Group C, year 2021, COVID-19^c^ (*n* = 78)	*P* value
Radiotherapy				0.758
Primary/definitive	49 (64.5%)	45 (63.4%)	46 (59.0%)	
Adjuvant	27 (35.5%)	26 (36.6%)	32 (41.0%)	
Intention of radiotherapy				0.054
Curative	66 (86.8%)	69 (97.2%)	73 (93.6%)	
Palliative	10 (13.2%)	2 (2.8%)	5 (6.4%)	

^a^Year 2019, pre-pandemic control group

^b^Year 2020, pandemic study group

^c^Year 2021, a phase of partial normalization in a persistent pandemic situation

Times to treatment initiation during the years 2019, 2020 and 2021 are presented in Table 4. For 179 patients, the surgery was conducted on the day of histological confirmation of cancer (year 2019, *n* = 67; year 2020, *n* = 56; year 2021, n = 56). Excluding these 179 patients, there was a decrease in median days between diagnosis and surgery that was significantly decreased in 2020 (19.5 days, IQR, 12.0–31.0; *P* = 0.049) and 2021 (20.0 days; IQR, 14.0–27.0; *P* = 0.026) in comparison to 2019 (23 days, IQR, 16.0–31.2). There were no differences regarding the median days between diagnosis and surgery between the years 2020 and 2021 (Table 4). The median days between diagnosis and definitive radiotherapy and the median days from surgery to adjuvant radiotherapy were not affected (Table 4).

**Table 4:** Time to treatment initiation during the years 2019, 2020 and 2021

	Group A, year 2019, pre-COVID-19^a^ (*n* = 186)	Group B, year 2020, COVID-19^b^ (*n* = 150)	Group C, year 2021, COVID-19^c^ (*n* = 191)	*P* value
Days from diagnosis^d^ to primary surgery Median (Interquartile range, IQR)	23.0 (16.0–31.2)^a^	19.5 (12.0–31.0)^b^	20.0 (14.0–27.0)^b^	0.013

	Group A, year 2019, pre-COVID-19^a^ (*n* = 49)	Group B, year 2020, COVID-19^b^ (*n* = 45)	Group C, year 2021, COVID-19^c^ (*n* = 46)	*P* value
Days from diagnosis^d^ to definitive radiotherapy Median (IQR)	33.0 (21.0–57.0)^a^	33.0 (22.0–54.0)^a^	27.5 (18.0–45.0)^a^	0.296

	Group A, year 2019, pre-COVID-19^a^ (*n* = 27)	Group B, year 2020, COVID-19^b^ (*n* = 26)	Group C, year 2021, COVID-19 (*n* = 32)	*P* value
Days from surgery to adjuvant radiotherapy Median (IQR)	37.0 (18.0–56.0)^a^	33.5 (19.0–57.0)^a^	33.0 (21.0–92.0)^a^	0.560

Groups with different letters (a, b) differ significantly at 5% significance level. Equal letters do not differ

^a^Year 2019, pre-pandemic control group

^b^Year 2020, pandemic study group

^c^Year 2021, a phase of partial normalization in a persistent pandemic situation

^d^Diagnosis, histological confirmation of cancer

## Discussion

The results presented did not show significant changes regarding T, N, and M classifications or UICC stages in patients presenting in 2020 and 2021 compared to those presenting in 2019. Other studies conducted on the possibility of upstaging in head and neck cancer patients presenting during the pandemic yielded heterogeneous results. Metzger et al. (2021) reported a significantly higher T classification in patients with oral cancer in 2020 compared to the years 2010–2019 whilst there were no significant changes regarding N classification or UICC stage. Tevetoğlu et al. (2021) also reported an increased rate of T3 and T4 tumors in 2020 compared to 2019. In this study an increase in N stage in 2020 was also reported, this finding however was limited to oral cavity cancers (Tevetoğlu et al. 2021). In another study patients presented with distant metastasis more frequently during the pandemic whilst T and N stages did not differ significantly compared to the pre-pandemic group (Kourtidis et al. 2022). Contrarily Balk et al. (2022) observed no significant difference regarding N stage, but noted a decrease in M1 stage during the defined COVID period. Our data revealed no significant decrease in head and neck cancer diagnoses in 2019, 2020, or 2021. Balk et al. (2022) using data of a large head and neck cancer center in Germany and comparing data from a COVID-19 period group (April 2020-April 2021) and pre-COVID-19 period group (March 2019-March 2020), found no decrease in the total number of patients with head and neck malignancies supporting the results of our study. Regarding the time between diagnosis and initiation of primary treatment, our study found a decrease in days to surgery that significantly decreased in 2020 and 2021 in comparison to 2019. Similarly, such a trend toward a decrease in time to intervention has been observed by Heimes et al. (2021) during the first lockdown between March and June 2020. Balk et al. (2022) reported no delay in the time to therapy during March 2019-March 2020 in comparison to April 2020-April 2021. Yao et al. (2021) found no significant difference in time to treatment between pre-COVID-19 and COVID-19 groups. Other studies have yielded differing results. Metzger et al. (2021) found significantly longer treatment delay in patients presenting in 2020 compared to patients presenting in 2010–2019. A time to treatment initiation (TTI) greater than 46–52 days and over 67 days has been demonstrated to carry an increased risk of mortality (Murphy et al. 2016). Furthermore, this increase in risk was greatest for patients in early stages of disease (Murphy et al. 2016). Considering our patient cohorts were made up of 45.0% (2019), 48.2% (2020), and 46.1% (2021) of patients with UICC stages I or II a relevant delay of TTI would pose an even more increased risk of mortality to nearly half of our cohorts. As demonstrated above the findings regarding time to treatment vary. We should also note the fact that some of the studies mentioned were conducted in other countries where different healthcare systems are implemented and affectation by COVID-19 may have differed from our region. The results of the present study indicate that the oncology care of head and neck cancer patients having received treatment at our head and neck cancer center was not negatively influenced by the pandemic. The significant increase in internally established diagnoses could be the result of direct referral to our head and neck cancer center for histological confirmation and treatment instead of prior referral to other institutions.

## Strengths and limitations

The limitations of the present study include the retrospective study design and the focus on a single head and neck cancer center. The present study analyzed treatment processes specifically at our facility and may not be indicative of other institutions as patients presenting at our head and neck cancer center usually show a high case complexity. Since our study included patients, whose malignancies were classified as non-primary we must acknowledge that in these cases patients may have presented earlier than those with primary malignancies or may have had their tumors diagnosed at follow-up visits for previous oncologic illnesses. Since the COVID-19 pandemic affected different geographical areas to different degrees, the results of this study are not necessarily indicative of the situation and the possible long-term effects in other areas of Germany. Nonetheless, the present study provides an important insight into the treatment of oncologic patients of a large head and neck cancer center throughout the first 2 years of the pandemic that may be helpful should similar situations arise in the future.

## Conclusions

The study revealed no significant change in the number and the stages of head and neck cancers in association with the COVID-19 pandemic in 2020 or the following year 2021 in comparison to 2019. The results indicate that the healthcare system of our head and neck cancer center was able to manage the oncologic care for head and neck cancer patients timely without delay.

## Data Availability

The datasets used and/or analyzed during the current study are available from the corresponding author on reasonable request.
